# Development of a highly tolerant bacterial consortium for asphaltene biodegradation in soils

**DOI:** 10.1007/s11356-023-30682-7

**Published:** 2023-11-20

**Authors:** Oscar Daniel Navas-Cáceres, Mayra Parada, German Zafra

**Affiliations:** Grupo de Investigación en Bioquímica y Microbiología (GIBIM), Escuela de Microbiología, Universidad Industrial de Santander, 680002 Bucaramanga, Colombia

**Keywords:** Asphaltenes, Biodegradation, Heavy crude oil, Bacterial consortium, Soil contamination, Tolerance

## Abstract

Asphaltenes are the most polar and heavy fraction of petroleum, and their complex structure and toxicity make them resistant to biodegradation. The ability to tolerate high asphaltene concentrations is crucial to reducing the toxicity-related inhibition of microbial growth and improving their capacity for adaptation, survival, and biodegradation in soils highly contaminated with asphaltenes. This study developed a highly tolerant consortium for efficient asphaltene biodegradation in soils from 22 bacterial isolates obtained from heavy-crude oil-contaminated soils. Isolates corresponded to the *Rhodococcus*, *Bacillus*, *Stutzerimonas*, *Cellulosimicrobium*, *Pseudomonas*, and *Paenibacillus* genera, among others, and used pure asphaltenes and heavy crude oil as the only carbon sources. Surface plate assays were used to evaluate the tolerance of individual isolates to asphaltenes, and the results showed variations in the extension and inhibition rates with maximum tolerance levels at 60,000 mg asphaltenes l^−1^. Inhibition assays were used to select non-antagonistic bacterial isolates among those showing the highest tolerance levels to asphaltenes. A consortium made up of the five most tolerant and non-antagonistic bacterial isolates was able to degrade up to 83 wt.% out of 10,000 mg asphaltenes kg^−1^ in the soil after 52 days. Due to its biological compatibility, high asphaltene tolerance, and ability to utilise it as a source of energy, the degrading consortium developed in this work has shown a high potential for soil bioremediation and is a promising candidate for the treatment of aged soil areas contaminated with heavy and extra-heavy crude oil. This would be the first research to assess and consider extreme bacterial tolerance and microbial antagonism between individual degrading microbes, leading to the development of an improved consortium capable of efficiently degrading high amounts of asphaltenes in soil.

## Introduction

Over the past few decades, the issue of petroleum-contaminated soil has gained widespread attention as a severe global environmental problem due to its toxicity and long-term persistence in the ecosystem. Human activities, including oil and gas exploration, transport, and refinement, as well as the improper management of hazardous oil waste, further increase the number of soil-polluted sites worldwide (Koshlaf and Ball 2017). Unconventional oil exploration has worsened the occurrence of this issue, as most crude oils produced by unconventional methods are heavy and extra-heavy and have elevated viscosities and densities. Heavy crude oils can have different amounts of light and heavy fractions, but they usually have less of the lighter aliphatic (saturated and unsaturated) hydrocarbons and more of the heavier aromatic, resin, and especially asphaltene compounds (Uche and Dadrasnia 2017). Because of its complex nature, asphaltene is the most polar and heaviest fraction of crude oil and could persist in the environment for decades, causing detrimental effects on contaminated soils, such as increased toxicity, decreased pore spaces, and alterations in electric conductivity, pH, texture, and bulk density (Brown et al. 2017). Bioremediation is an economically viable and environmentally sustainable approach for addressing soil contamination caused by heavy hydrocarbons such as asphaltenes. However, various abiotic and biotic factors have a significant impact on the effectiveness of bioremediation. These factors include the concentration, bioavailability, and toxicity of the pollutants, as well as the physicochemical characteristics of the soil, such as pH, texture, moisture content, nutrient availability, and oxygen levels. The presence and metabolic potential of microorganisms capable of degrading contaminants, as well as the interactions between indigenous and introduced microorganisms, also play a significant role in determining the success of bioremediation efforts (Al-Hawash et al. 2018). Asphaltenes are poorly volatile, almost insoluble in both water and organic solvents, and, subsequently, scarcely available for microbial degradation (Chuah et al. 2023). Bioremediation of aged soils contaminated for long periods represents a challenge as the heaviest fractions of petroleum strongly bind to soil organic matter, reducing their availability for microorganisms even more (Chuah et al. 2022). Even though different microorganisms have been reported to partially degrade asphaltenes (Lavania et al. 2012) and even use them as their only carbon source (Pineda-Flores et al. 2004), their biotransformation into smaller and less toxic compounds is likely to be the result of a synergistic action between different microorganisms due to the complex structure and chemical characteristics of asphaltenes (Tavassoli et al. 2012). The use of microbial consortia for soil bioremediation is a convenient strategy, as probably no single microbial species could metabolise all the crude oil fractions (Hii et al. 2009). Several studies have reported the use of microbial consortia as an effective approach for the biodegradation of pure asphaltenes; however, due to the toxicity and low bioavailability of asphaltenes, their degradation rate is typically low (Jahromi et al. 2014), and most of these studies have been conducted using only liquid cultures, not contaminated soils (Shahebrahimi et al. 2020; Yanto and Tachibana 2014a).

Besides the innate ability of a microbial consortium to partially or completely metabolise asphaltenes, a viable soil remediation technology requires microorganisms capable of rapid adaptation and permanence. The use of microbes tolerant to high amounts of asphaltenes would be desirable to minimise toxicity-mediated microbial growth inhibition and presumably improve their adaption, permanence, and degradation potential in highly contaminated soils (Montgomery et al. 2010). In that sense, the use of rigorous methods to obtain, characterise, and select novel native microbes with the ability not only to tolerate and degrade extreme concentrations of asphaltenes but also to have biological compatibility to establish interspecific cooperations would be key factors for the successful development of consortia that could be used efficiently under different conditions to mitigate asphaltene pollution in soils. This work aimed to isolate, characterise, and select native bacterial isolates with very high levels of asphaltene tolerance, wide catabolic potential, compatibility, and the capability of mineralizing asphaltene fractions to develop for the first time a highly tolerant bacterial consortium able to efficiently degrade asphaltene fractions on highly polluted soils.

## Materials and methods

### Materials

All the solvents used for hydrocarbon preparation, spiking, and extraction from soil (dichloromethane, methanol, and toluene) were analytical grade and provided by Sigma-Aldrich. Heavy crude oil and asphaltene samples (molecular composition of C: 84%; H: 8%; N: 1.5%; S: 4.5%; O: 1.5%; H/C ratio: 1.14; Ni: 406 mg kg^−1^, V: 1601 mg kg^−1^, MW: 2000 Da) were provided by the Colombian Institute of Petroleum. M9 minimal medium used for microbial isolation was composed of Na_2_HPO_4_, 12.8 g l^−1^; KH_2_PO_4_, 3 g l^−1^; NH_4_Cl, 1 g l^−1^; CaCl_2_.2H_2_O, 0.015 g l^−1^; FeSO_4_.6H_2_O, 0.01 g l^−1^ (Sigma-Aldrich); NaCl, 0.5 g l^−1^; and MgSO_4_.7H_2_O, 1 g l^−1^ (Carlo Erba Reagents) (Cold Spring Harbor Laboratory 2010). Starch casein agar (SCA) was composed of starch, 10 g l^−1^; casein, 0.3 g l^−1^ (Scharlau); KH_2_PO_4_, 2 g l^−1^; NH_4_Cl, 2 g l^−1^; FeSO_4_.6H_2_O, 0.05 g l^−1^; FeSO_4_.6H_2_O, 0.01 g l^−1^; and bacteriological agar, 15 g l^−1^ (Sigma-Aldrich) (Kuster and Williams 1964). Brain heart infusion (BHI) broth was composed of brain heart infusion, 17.5 g l^−1^; proteose peptone, 10 g l^−1^; NaCl, 5 g l^−1^; Na_2_HPO_4_, 2 g l^−1^; and dextrose, 2 g l^−1^ (PanReac AppliChem).

### Soil samples

Aged heavy crude oil-contaminated soil samples were obtained from the Lisama oil field in Santander, Colombia (7°05′39.7″N; 73°33′29.7″W). Composite samples of 100 g each were collected randomly at a depth of 30 cm, following the sampling procedures described by U.S. EPA (US-EPA 1996). Uncontaminated soil samples were obtained from the same coordinates. Once collected, samples were refrigerated at 4 °C until processing.

### Isolation and identification of crude oil-degrading bacteria from soil

Native bacterial isolates were obtained by diluting 1 g of soil in 9 ml of liquid M9 minimal medium supplemented with 0.1 wt.% heavy crude oil (API gravity of 17.8°) as the sole carbon source. Cultures were incubated at 30 °C for 48 h under constant shaking at 200 g. Subsequently, 100 μl of the culture supernatants was inoculated into plates of solid M9 medium supplemented with 0.1 wt.% heavy crude oil and incubated at 30 °C until visible growth was observed. Colonies were picked and transferred into new plates of M9 medium with heavy crude oil as a carbon source. Actinomycetes were isolated using Starch Casein Agar following the same methodology described above.

Genomic DNA extractions from each pure isolate were performed using the Wizard Genomic DNA Purification Kit (Promega). DNA quantity and quality were measured by agarose gel electrophoresis and by the ratio of absorbance at 260 nm and 280 nm. Amplification of a 1450-bp fragment of the bacterial 16S rRNA gene was performed using primers P27F (GAGTTTGATCCTGGCTCA) and P1525R (GAAAGGAGGAGATCCAGC) (Lane 1991). Amplification conditions consisted of an initial denaturation at 95 °C for 5 min, followed by 35 cycles of 95 °C for 45 s, 58 °C for 30 s, 72 °C for 75 s, and a final extension at 72 °C for 10 min. PCR products were purified using the QIAquick PCR purification kit (QIAGEN) and sequenced in a 3730xl DNA analyzer (Applied Biosystems), using oligonucleotides P27F and P1525R as sequencing primers. The web-based basic local alignment tool (nucleotide BLAST) was used to compare ribosomal sequences with the GenBank database for microbial identification (Altschul et al. 1990). Phylogenetic analysis was performed using the neighbor-joining algorithm as implemented in MEGA11 (Tamura et al. 2021).

### Microbial tolerance tests to asphaltenes

Surface plate assays were used to determine each bacterial isolate’s tolerance to progressively higher concentrations of pure asphaltenes (Zafra et al. 2014). Each isolate was grown in 5 ml of BHI broth at 30 °C with agitation until cultures reached an optical density of 0.08 at 600 nm (comparable to a McFarland standard No. 0.5). One hundred microliters of each culture (approximately 1.5 × 10^7^ colony forming units (CFU)) were spotted as drops over the surface of BHI plates containing pure asphaltenes at final concentrations of 100, 600, 1000, 3000, 8000, 10,000, 20,000, and 60,000 mg l^−1^. Plates were incubated at 30 °C for up to 7 days. BHI plates without asphaltenes and inoculated with each of the isolates were used as growth controls. Isolates showing visible growth after incubation were considered tolerant to the corresponding dose of asphaltenes in media.

### Evaluation of bacterial antagonism

Inhibition surface assays were used to detect microbial antagonism between the isolates showing the highest tolerance levels to asphaltenes (Zafra et al. 2017). Each of the asphaltene-tolerant isolates was grown in 10 ml of BHI broth at 30 °C to reach an optical density of 0.08 at 600 nm. Approximately 1.5 × 10^7^ CFU of each culture (100 µl) were massively spread over the surface of individual BHI plates, allowed to dry for 5 min, and then 10 µl of each of the other isolates were spotted as drops over the medium surface. Plates were incubated at 30 °C for 24 h or until the formation of a bacterial lawn. A lack of growth in the spotted isolates or the presence of inhibition halos from the spotted isolates towards the bacterial lawn of the massively inoculated isolate was considered a signal of microbial antagonism.

### Definition of the asphaltene-degrading bacterial consortium by self-selection in soil

Only bacterial isolates tolerant to 60,000 mg asphaltenes l^−1^ and showing no antagonistic effects towards other tolerant isolates were considered for the construction of a degrading consortium. An induced natural selection method using solid culture systems was used to establish an optimal isolate combination and test the consortium’s survival in asphaltene-contaminated soils using biotic (microbial interactions between introduced isolates) and abiotic (pure asphaltenes) selective factors (Zafra et al. 2017). Solid culture systems were prepared in 100-ml glass reactors containing 6.65 g of sterile soil previously spiked with 5000 mg of asphaltenes kg^−1^, 0.35 g of sterile sugarcane bagasse as a soil texturizing amendment, and inoculated with 2 × 10^6^ CFU g^−1^ of each bacterial isolate. Inoculated reactors were hermetically sealed and incubated at 30 °C for 30 days, flushing headspaces every 48 h for 15 min with sterile and moistened air to preserve aerobic conditions and avoid carbon dioxide accumulation. Microbial survival in soil was detected by direct isolation on BHI plates after 30 days. Low-stringency single-specific primer PCR (LSSP-PCR) was used to detect isolate-specific DNA band profiles after 30 days of culture. Total genomic DNA was extracted from 0.25 g soil samples using the DNeasy PowerSoil Pro Kit (QIAGEN). LSSP-PCR reactions were carried out by purifying 16S rRNA gene 1450 bp amplicons obtained from the soil with the primer set P27F/1525R and performing a second round of amplification using only the 27F primer under conditions of low stringency. Reactions were performed in a final volume of 20 µl containing 3 U of Taq DNA polymerase (Invitrogen), 200 μM dNTPs, 3 mM MgCl_2_, and 5 μM of primer P27F. The amplification conditions were as follows: an initial denaturation at 95 °C for 5 min, then 30 cycles of 95 °C for 30 s, 34 °C for 60 s, and 72 °C for 60 s. The isolates chosen to be included in the degrading consortium resulting from this approach would be those with the highest tolerance levels to asphaltenes, exhibiting no antagonistic effects towards other isolates and having been either cultured or detected through LSSP-PCR in contaminated soils after a period of 30 days. All assays were carried out in triplicate. Selected bacterial isolates were further subjected to biochemical characterisation with the RapID™ CB PLUS and RapID™ NF PLUS systems (Thermo Scientific) to test their degradation abilities of specific substrates (carbohydrates, aryl C-glycosides, lipids, and amino acids).

### Asphaltene degradation by the bacterial consortium in soil

The asphaltene degradation ability of the consortium was measured in solid culture reactors containing 6.65 g of non-sterile soil spiked with 10,000 mg of asphaltenes kg^−1^, 0.35 g of sterile sugarcane bagasse as a soil texturizing and stimulating agent for native microbiota, and 1 × 10^7^ CFU g^−1^ of the bacterial consortium (biostimulation plus bioaugmentation). The ratio of soil to agro-industrial waste was 95:5. All reactors were aerated with sterile and moistened air for 15 min every 48 h to preserve aerobic conditions and incubated at 30 °C for 52 days. Reactors containing contaminated soil and sugarcane bagasse but not inoculated with the consortium were used to measure the degradation rates of soil native microbiota (biostimulation only). Controls, consisting of non-contaminated soil treated by bioaugmentation and biostimulation, were included to determine the soil basal respiratory levels in the absence of asphaltenes. Bacterial respiratory activity was measured by the amount of carbon dioxide (CO_2_) produced every 48 h in each of the reactors using a 7752 AZ CO_2_ detector (AZ Instruments Corp.) equipped with a non-dispersive infrared sensor. CO_2_ was reported as mg of CO_2_ per g of initial dry matter (soil plus sugarcane bagasse). The extraction of residual asphaltenes from soil was performed by the shaking-centrifugation method, as reported by Arce-Ortega et al. (2004), using a 2:1 mixture of dichloromethane and methanol as the extracting solvent. The degradation of asphaltenes in soil was measured by gravimetry according to the ASTM D6560 standard method (ASTM 2022). All assays were carried out in triplicate.

### Statistical analysis

Data from CO_2_ measurements and asphaltene biodegradation were analysed by one-way analysis of variance (ANOVA) followed by a Tukey multiple comparison test with SPSS Statistics Software version 20 (IBM). A *p*-value of < 0.05 was considered statistically significant.

## Results

### Microbial isolation from soil

**Fig. 1:**
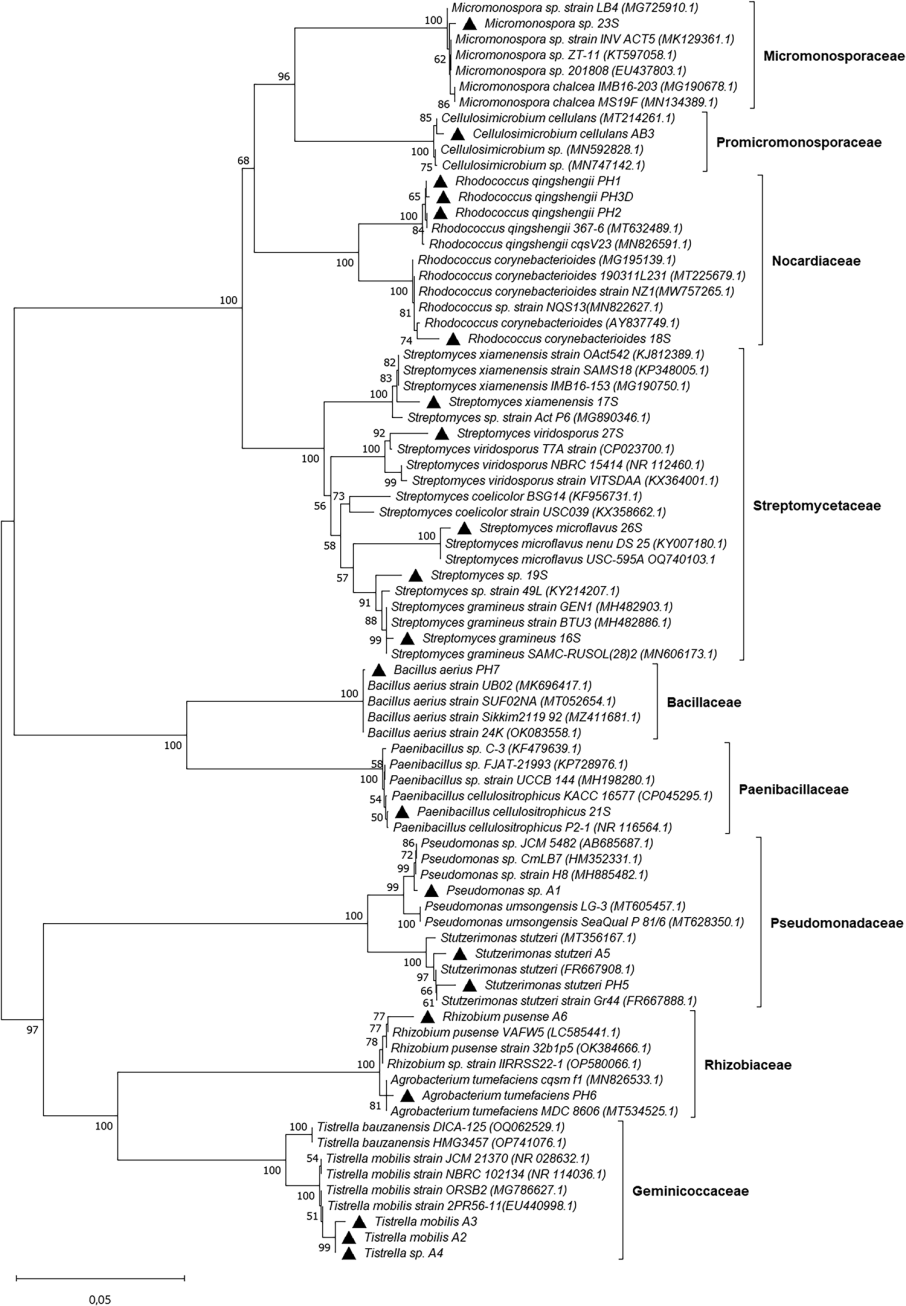
Phylogenetic tree showing the clustering and similarities of bacterial isolates based on 16S rRNA gene sequences. Analysis was performed using the neighbor-joining algorithm with the Kimura two-parameter model. Numbers on the nodes denote percent bootstrap values based on 1000 replicates. Black triangles indicate the sequences corresponding to organisms isolated in this study. GenBank accession numbers of reference sequences are indicated in brackets

Twenty-two bacterial isolates were obtained from heavy crude oil-contaminated soils based on their capacity to tolerate and use heavy crude oil as the sole carbon and energy source. Morphological analysis of bacterial isolates showed diversity in macroscopic and microscopic morphologies, including Gram-positive cocci, Gram-negative and positive bacilli, and actinobacterial filaments. The 16S rRNA gene sequence analysis indicated that isolates were phylogenetically diverse (Fig. 1) and distributed among nine families and 11 different bacterial genera (*Cellulosimicrobium*, *Rhodococcus*, *Paenibacillus*, *Pseudomonas*, *Stutzerimonas*, *Agrobacterium*, *Bacillus*, *Tistrella*, *Rhizobium*, *Streptomyces*, and *Micromonospora*) with genetic similarity values close to 100% with those reported in GenBank (Table 1). Sequences from isolates belonging to the same species (e.g., *Rhodococcus qingshengii*, *Stutzerimonas stutzeri*, *Tistrella mobilis*) were found not to be 100% similar among them.

**Table 1:** BLAST identification of bacterial isolates obtained from heavy crude oil-contaminated soils based on 16S rRNA sequences

Isolate	Species	Identity	Phylum	Family
A1	*Pseudomonas* sp.	99%	Pseudomonadota	Pseudomonadaceae
A2	*Tistrella mobilis*	100%	Pseudomonadota	Geminicoccaceae
A3	*Tistrella mobilis*	99.4%	Pseudomonadota	Geminicoccaceae
A4	*Tistrella* sp.	100%	Pseudomonadota	Geminicoccaceae
A5	*Stutzerimonas stutzeri*	99%	Pseudomonadota	Pseudomonadaceae
A6	*Rhizobium pusense*	98%	Pseudomonadota	Rhizobiaceae
AB3	*Cellulosimicrobium cellulans*	99%	Actinomycetota	Promicromonosporaceae
PH1	*Rhodococcus qingshengii*	99%	Actinomycetota	Nocardiaceae
PH2	*Rhodococcus qingshengii*	99%	Actinomycetota	Nocardiaceae
PH3	*Agrobacterium* sp.	99%	Pseudomonadota	Rhizobiaceae
PH3D	*Rhodococcus qingshengii*	99%	Actinomycetota	Nocardiaceae
PH5	*Stutzerimonas stutzeri*	99.6%	Pseudomonadota	Pseudomonadaceae
PH6	*Agrobacterium tumefaciens*	98%	Pseudomonadota	Rhizobiaceae
PH7	*Bacillus aerius*	99%	Bacillota	Bacillaceae
16S	*Streptomyces gramineus*	99%	Actinomycetota	Streptomycetaceae
17S	*Streptomyces xiamenensis*	98%	Actinomycetota	Streptomycetaceae
18S	*Rhodococcus corynebacterioides*	99%	Actinomycetota	Nocardiaceae
19S	*Streptomyces* sp.	100%	Actinomycetota	Streptomycetaceae
21S	*Paenibacillus cellulositrophicus*	100%	Bacillota	Paenibacillaceae
23S	*Micromonospora* sp.	98%	Actinomycetota	Micromonosporaceae
26S	*Streptomyces microflavus*	99%	Actinomycetota	Streptomycetaceae
27S	*Streptomyces viridosporus*	99%	Actinomycetota	Streptomycetaceae

### Tolerance of bacterial isolates to asphaltenes

**Fig. 2:**
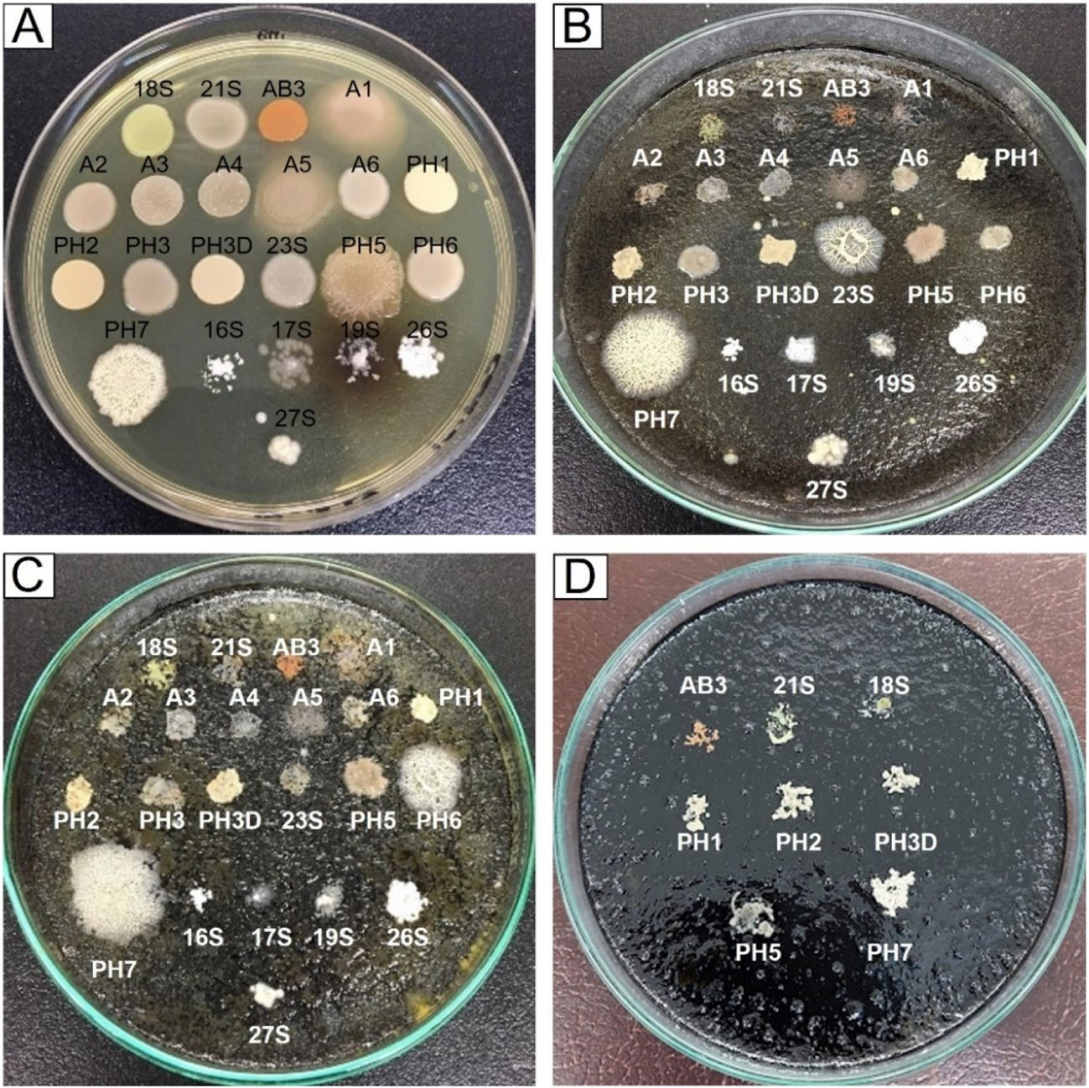
Tolerance tests of 22 bacterial isolates to pure asphaltenes after 48 h growth. Eight individual isolates were able to grow at a concentration of 60,000 mg asphaltenes l^−1^. Representative pictures showing the growth of isolates on BHI medium containing **A** no asphaltenes, **B** 2000, **C** 6000, and **D** 60,000 mg asphaltenes l.^−1^

All the isolated bacteria showed visible growth after 7 days in BHI plates containing 100, 600, 1000, 3000, 8000, and 10,000 mg of asphaltenes l^−1^, and only eight isolates were able to tolerate 20,000 and 60,000 mg of asphaltenes l^−1^ (Fig. 2). These eight bacterial isolates (AB3, PH1, PH2, PH3, PH5, PH7, 18S, and 21S) were subjected to further steps in the development of the degrading microbial consortium.

### Definition of the asphaltene-degrading microbial consortium

**Fig. 3:**
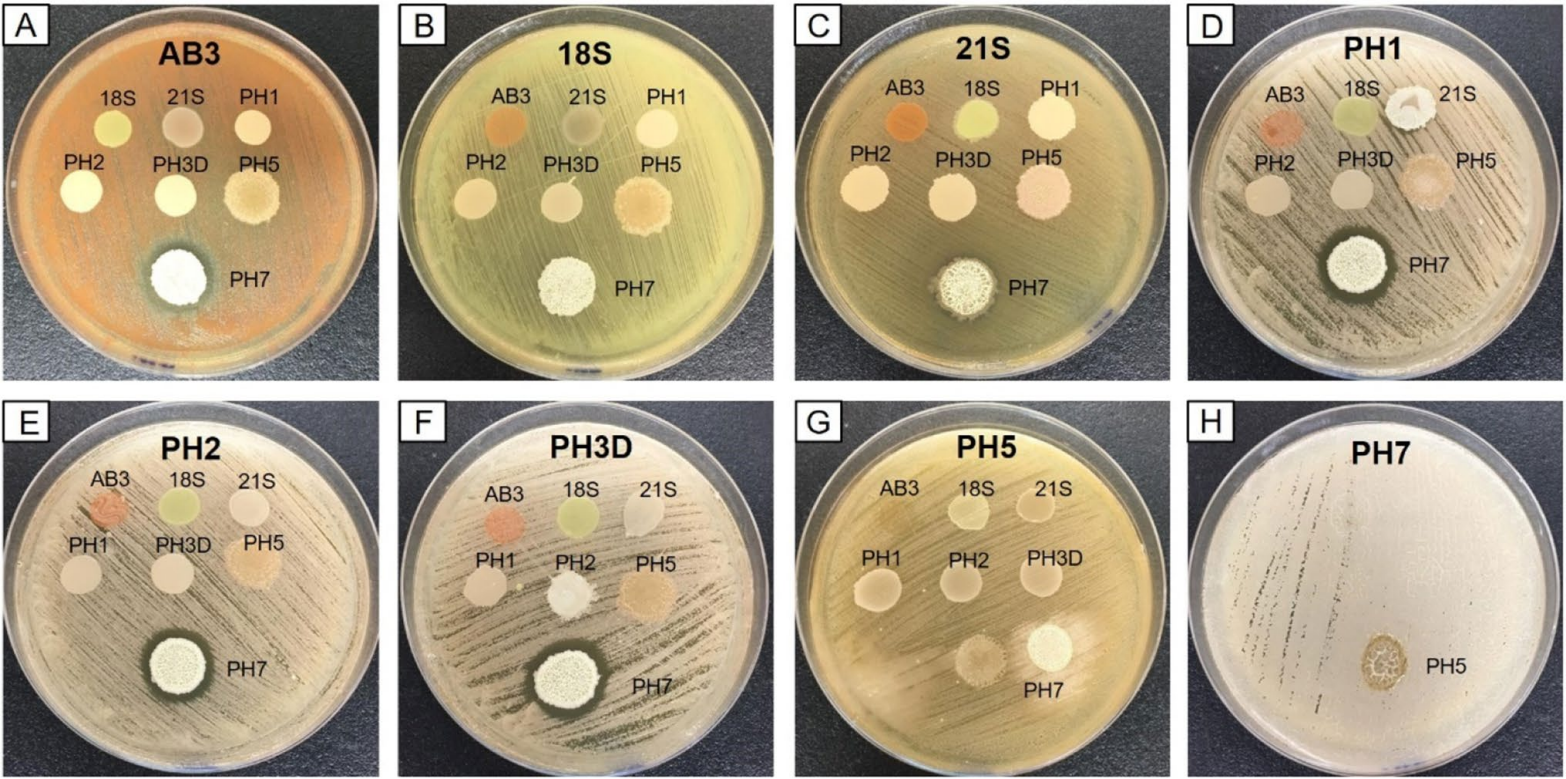
Growth inhibitory effects between asphaltene-tolerant bacterial isolates. The bacterial lawn of isolates AB3 (**A**), 18S (**B**), 21S (**C**), PH1 (**D**), PH2 (**E)**, PH3 (**F**), PH5 (**G**), and PH7 (**H**) were tested against the remaining isolates spotted as drops on BHI plates. Only isolate PH7 (*Bacillus aerius*) showed antagonism towards six of the other bacterial isolates

**Fig. 4:**
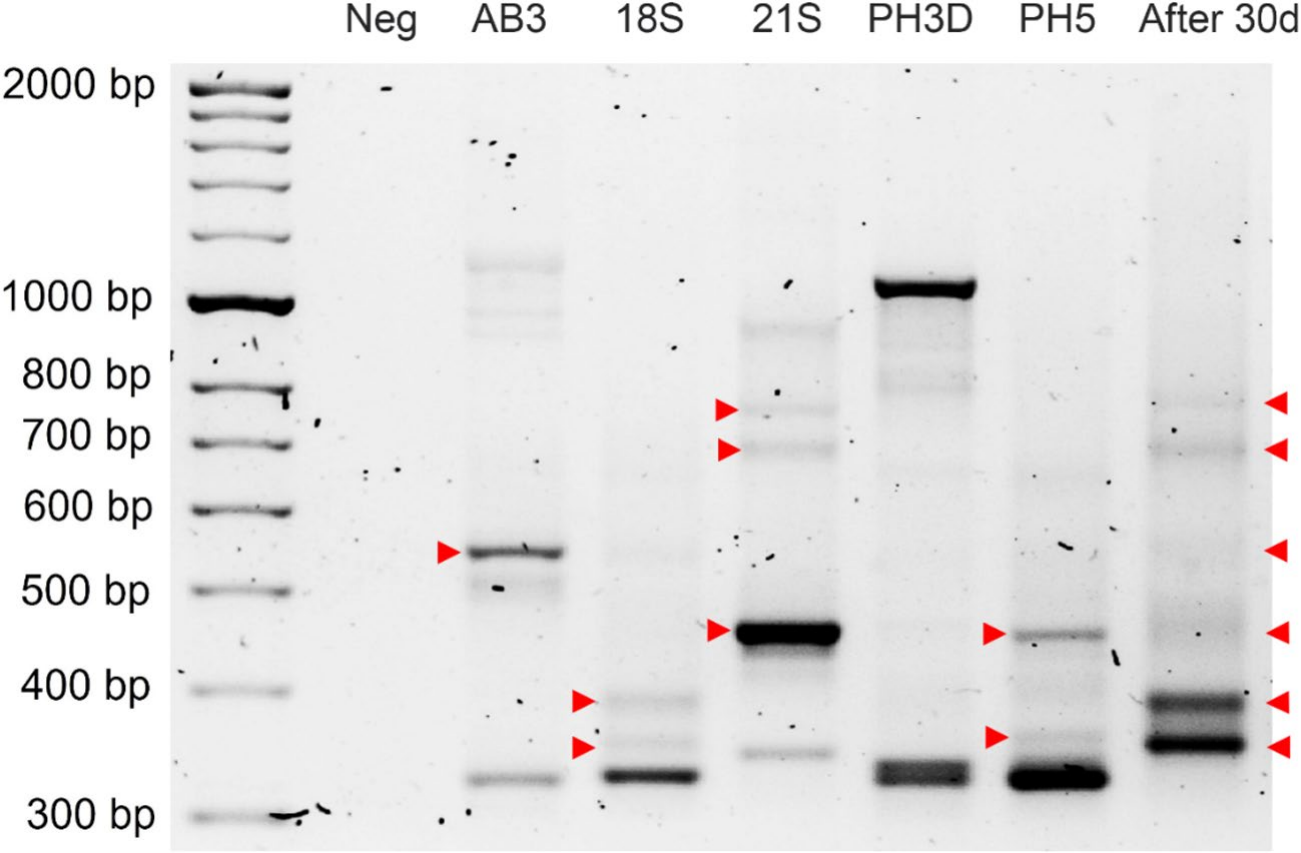
LSSP-PCR profiles from individual bacterial isolates and soil contaminated with 5000 mg asphaltenes kg^−1^ and inoculated with a combination of isolates AB3, 18S, 21S, PH3D, and PH5. Red arrows indicate the individual bands remaining after 30 days of incubation, which along with microbial isolation on BHI agar, allowed to confirm the permanence of all bacterial isolates in soil. Neg, negative control

Among the eight isolates tolerant to 60,000 mg asphaltenes l^−1^, only isolate PH7 (*Bacillus aerius*) was antagonistic to six of the other isolates (Fig. 3). In addition to being inhibited by isolate PH7, isolates PH1 and PH2 (*Rhodococcus qingshengii*) presented slow growth rates when cultured on media with and without asphaltenes. Except for these three mentioned isolates, the results indicated that the five remaining bacterial isolates did not produce or suffer any antagonistic effect, had comparable growth rates, and were suitable to construct a degrading consortium with no major antagonistic effects between them. When a mixture of isolates AB3, 18S, 21S, PH3D, and PH5 was inoculated into soil contaminated with 5000 mg of asphaltenes kg^−1^, culture isolation showed that all five of the introduced bacteria survived and prevailed after 30 days. In addition, except for the PH3D isolate, partial LSSP-PCR band profiles from all bacterial isolates were detected after 30 days, (Fig. 4). Considering the results from culture isolation and LSSP-PCR, the five tolerant bacterial isolates (*Cellulosimicrobium cellulans* AB3, *Rhodococcus corynebacterioides* 18S, *Paenibacillus cellulositrophicus* 21S, *Rhodococcus qingshengii* PH3D, and *Stutzerimonas stutzeri* PH5) were selected to compose the asphaltene-degrading microbial consortium. 16S rRNA gene partial sequences from these isolates were deposited in GenBank under accession numbers MW261332, MW261333, MW261334, MW261335, and MW261336. Biochemical characterisation of the isolates showed a broad range of substrate utilisation (Table 2). All the isolates were able to hydrolyse fatty acid esters and a variety of carbohydrate and amino acid substrates, with a predominance of fermentative carbohydrate metabolism.

**Table 2:** Substrate utilisation by bacterial isolates composing the asphaltene-degrading consortium

Substrate	Isolate				
	*Paenibacillus cellulositrophicus* 21S	*Rhodococcus corynebacterioides* 18S	*Cellulosimicrobium cellulans* AB3	*Rhodococcus qingshengii* PH3D	*Stutzerimonas stutzeri* PH5
Glucose	+	+	+	+	+
Sucrose	−	+	+	−	−
Ribose	+	+	+	+	NE
Maltose	+	+	+	−	NE
Aliphatic thiol	NE	NE	NE	NE	+
αGLU	+	+	+	+	+
βGLU	+	+	+	+	−
NAG	+	+	−	−	−
GLY1	−	−	−	−	−
ONPG	−	+	−	−	−
PHS	+	−	+	+	+
FAE	+	+	+	+	+
Triglyceride	NE	NE	NE	NE	+
PRO	−	+	+	+	+
TRY	+	+	+	+	+
PYR	+	+	−	−	−
LGLY	+	+	+	+	NE
LEU	+	+	+	+	NE
GGT	NE	NE	NE	NE	+
BANA	NE	NE	NE	NE	+
Urea	−	−	−	+	−
Nitrate reduction	−	+	−	−	−

+ , positive; − , negative; *NE*, not evaluated; *αGLU*, ρ-nitrophenyl-α, D-glucoside; *βGLU*, ρ-nitrophenyl-β,D-glucoside; *NAG*, ρ-nitrophenyl-n-acetyl-β, D-glucosaminide; *GLY1*, ρ-nitrophenyl-glycoside; *ONPG*, σ-nitrophenyl-β,D-galactoside; *PHS*, ρ-nitrophenyl phosphate; *FAE*, fatty acid ester; *PRO*, proline-β-naphthylamide; *TRY*, tryptophan-β-naphthylamide; *PYR*, pyrrolidine-β-naphthylamine; *LGLY*, leucyl-glycine-β-naphthylamide; *LEU*, leucine-β-naphthylamide; *GGT*, ɣ-glutamyl β-naphthylamide; *BANA*, N-benzyl-arginine-β-naphthylamide

### Growth and degradation of asphaltenes by the bacterial consortium in contaminated soil

**Fig. 5:**
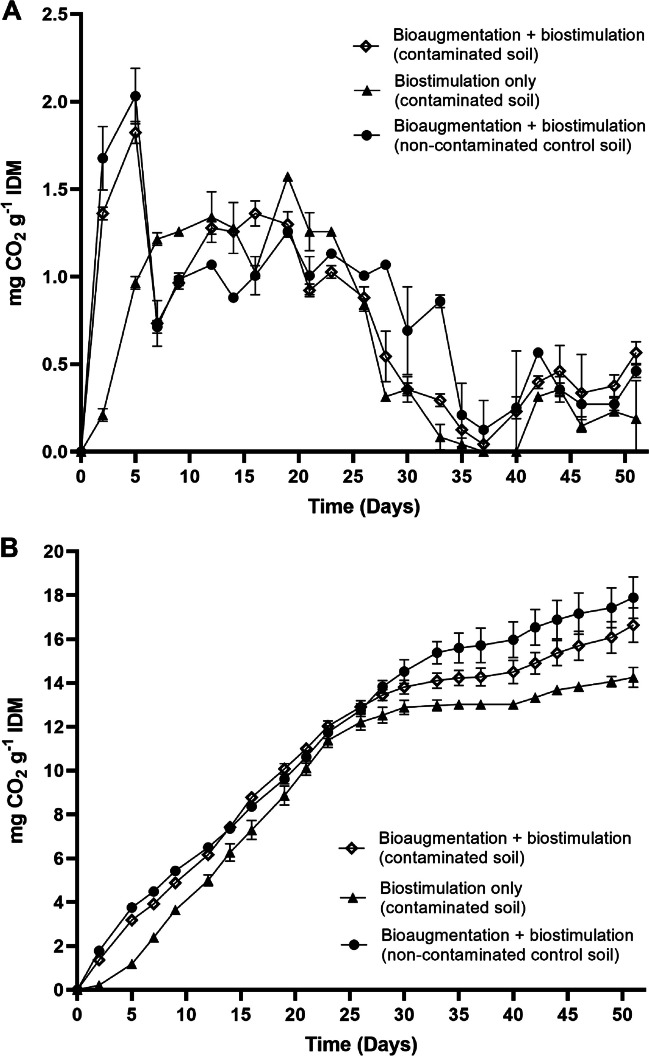
Instantaneous (**A**) and accumulated (**B**) CO_2_ production in soils contaminated with 10,000 mg asphaltenes kg^−1^ and inoculated with the bacterial consortium during a 52-day treatment. Asphaltene-contaminated soils inoculated with the consortium and biostimulated (bioaugmentation + biostimulation) produced a significantly higher amount of CO_2_ at day 52 than the asphaltene-contaminated soils that were only biostimulated. Error bars represent standard errors of the means (*n* = 3). IDM, initial dry matter

**Fig. 6:**
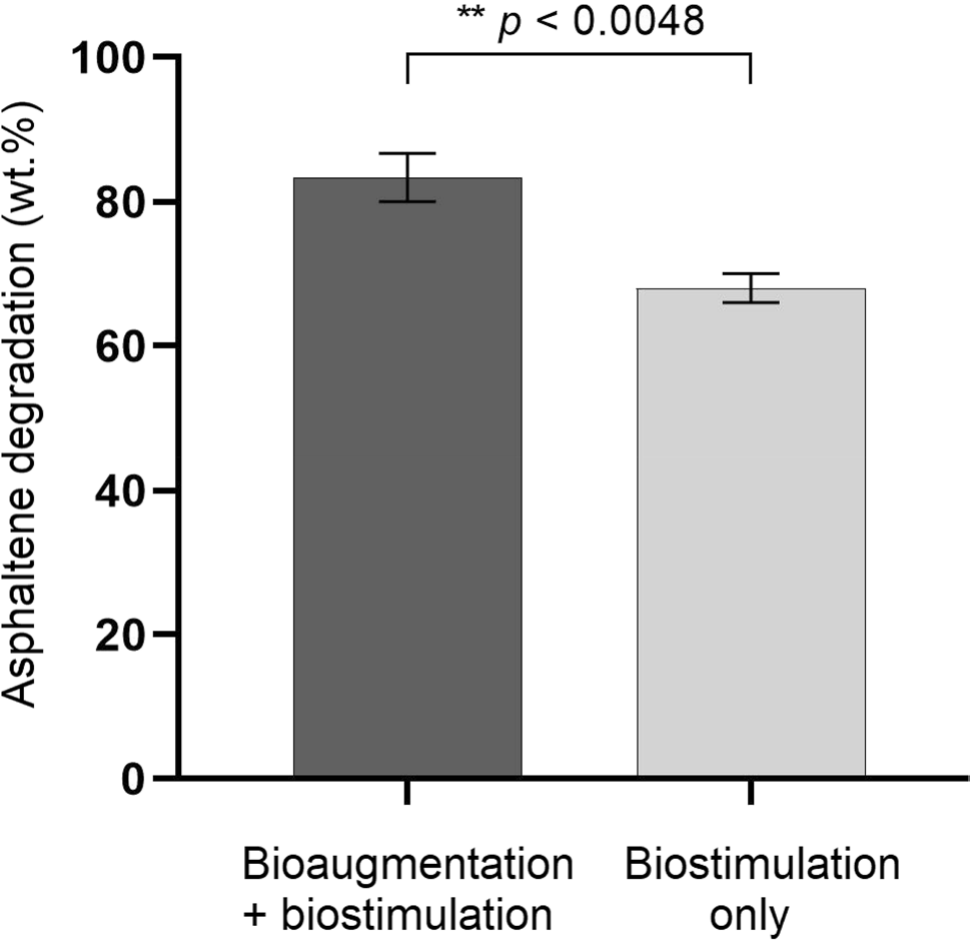
Asphaltene degradation in soil contaminated with 10,000 mg asphaltenes kg.^−1^ after a 52-day treatment. Degradation efficiencies were significantly different between the two treatments. Error bars represent standard errors of the means (*n* = 3)

The rates of CO_2_ production through the 52-day degradation process indicated sustained microbial activity in both contaminated and uncontaminated soils, either inoculated or not with the consortium. The maximum CO_2_ production was obtained in non-contaminated control soils as a reflection of the lack of growth inhibition caused by asphaltenes. The presence of asphaltenes in soil induced a rapid initial increase in CO_2_ production by microbial-degrading populations during the first week of treatment (Fig. 5). Asphaltene-contaminated soils bioaugmented with the bacterial consortium and biostimulated produced a significantly higher amount of CO_2_ at day 52 than the asphaltene-contaminated soils that were not inoculated and only biostimulated (*p* = 0.001). This difference correlated with asphaltene degradation efficiency, as bioaugmented and biostimulated soils reached up to 83 wt.% degradation out of 10,000 mg of asphaltenes kg^−1^ after 52 days, in comparison to 63 wt.% achieved when only biostimulation was used (*p* = 0.0048) **(**Fig. 6).

## Discussion

Although many hydrocarbon-degrading microorganisms have been described, only a few studies have explored how microbial tolerance to heavy hydrocarbons affects bioremediation performance. Asphaltenes are harmful and toxic organic molecules that could serve as a carbon and energy source for microbial communities in hydrocarbon-contaminated soils. Poor tolerance of microbial populations to toxic hydrocarbons, such as asphaltenes, may have a significant impact on the persistence of petroleum-heavy fractions in soils. Consequently, bioremediation strategies on these soils perform suboptimally due to decreased microbial adaptation, inhibited growth, and overall catabolic repression (Montgomery et al. 2010).

This study focused on isolating and characterising heavy hydrocarbon-degrading bacterial isolates with extreme tolerance levels to asphaltenes to develop a microbial consortium able to degrade asphaltenes at high rates on severely contaminated soils. A variety of native bacterial species were isolated from an aged, heavy crude oil-contaminated soil. Most of them have already been described as capable of using heavy crude oil or asphaltene as the sole carbon source. Even though isolates of several bacterial genera studied in this work have already been described as hydrocarbon-degrading microbes, such as *Pseudomonas* (Yetti et al. 2016), Bacillus (Mansur et al. 2014), Paenibacillus (Mesbaiah et al. 2016), Tistrella (Zhang et al. 2011), Rhodococcus, and Streptomyces (Ubani et al. 2022), among others, the variety of microbial species capable of degrading and tolerating high concentrations of pure asphaltenes has been much less reported. To our knowledge, among the isolated species in this study, only Rhodococcus qingshengii has been reported to mineralise pure asphalt to some extent (Nie et al. 2021). Only eight out of 22 of the native bacterial isolates described in this study were able to tolerate very high concentrations of 20,000 and 60,000 mg asphaltenes l^−1^, which would be equivalent to a 2 and 6 wt.% content in soil, respectively, indicating that not all soil native bacterial populations can withstand high amounts of asphaltenes when present in real contaminated soils or are able to actively grow and mineralise them under such adverse conditions.

Perhaps one of the main reasons to use microbial consortia for bioremediation purposes lies in achieving a synergistic, complementary, and cometabolic degradation of contaminants. This is especially important for asphaltene biodegradation, as it is a complex mixture of aromatic and non-aromatic rings associated with alkyl side chains of heteroatoms with no specific degradation mechanisms yet demonstrated (Nzila and Musa 2021). Most studies about asphaltene biodegradation by microbial consortia used either liquid mineral media supplemented with asphaltenes or soils containing asphaltenes at concentrations below those used in this study. Using liquid media, for instance, Jahromi et al. (2014) reported degradation of 51.5 v.% out of 35,000 mg asphaltenes l^−1^ after 60 days using consortia composed of isolates of *Pseudomonas*, *Citrobacter*, *Enterobacter*, *Bacillus*, and *Lysinibacillus*; Kshirsagar et al. (2020) achieved a 35 v.% asphaltene removal after 15 days in media containing 10,000 mg of asphaltenes l^−1^ using a consortium composed of six bacterial isolates belonging to *Pseudomonas*, *Bacillus*, *Lysinibacillus*, and *Ochrobacter*; and Shahebrahimi et al. (2020) described a degradation of 46.4 v.% out of 35,000 mg asphaltenes l^−1^ in liquid media after 60 days by a consortium composed of *Staphylococcus saprophyticus* and *Bacillus cereus.* Yanto and Tachibana (2014b), on the other hand, reported a maximum degradation efficiency of 33.3 wt.% out of 4200 mg of asphaltenes kg^−1^ soil after 30 days using a fungal consortium of *Pestalotiopsis* sp. and *Polyporus* sp. Considering the results from these studies, it is clear that (1) efficient asphaltene biodegradation in the soil is more difficult to achieve than in liquid media supplemented with partially dissolved asphaltenes; (2) degradation efficiency strongly depends on asphaltene concentrations in the contaminated matrix and treatment duration; and (3) degrading microbial consortia must be developed considering their tolerance to asphaltenes, biological compatibility, catabolic potential, successful adaptation, and long-term survival in soil. These factors could determine the overall efficiency of the consortium in a highly contaminated environment and be key to promoting cometabolism, as functional metagenomic analyses have demonstrated in high-molecular-weight polycyclic aromatic hydrocarbon (PAH)-contaminated soils (Zafra et al. 2016).

Unlike other studies, this work considered all the factors above to develop a degrading consortium based on their high tolerance to asphaltenes, the ability to use them as the sole carbon source, lack of antagonism, prolonged survival in soil, and reported capacity to metabolise asphaltenes. The five bacterial isolates composing the consortium presented different patterns of substrate utilisation and were able to use lipids as growth support, especially fatty acid esters, as well as amino acid derivates, which reflects their metabolic potential and could confer an advantage in terms of both adaptation and biodegradation efficiency. Previous studies have explored the ability to produce aliphatic and aromatic hydrocarbon-degrading enzymes by the bacterial species conforming the consortium, e.g., hemicellulase, exoglycanases, xylanases, and laccases from *Cellulosimicrobium cellulans* (Wang et al. 2018), alkane and cytochrome P450 monooxygenases, ring-hydroxylating dioxygenases, laccases and dehalogenases from *Rhodococcus corynebacteroides* (Chukwuma et al. 2021) and *Rhodococcus qingshengii* (Lincoln et al. 2015), ligninase and cellulase from *Paenibacillus cellulositrophicus* (Rohman et al. 2015), and alkane hydroxylase and laccase from *Stutzerimonas stutzeri* (Parthipan et al. 2017). The results presented here showed that *Stutzerimonas stutzeri* PH5 is also capable of using aliphatic sulphur compounds (aliphatic thiols and mercaptans) and potentially other reduced sulphur-containing compounds as a source of energy, which have been identified as linking structures in asphaltene and resin alkyl bridges (Bava et al. 2019). Biocatalytic cleavage of these carbon–sulphur bonds has been reported to produce a fourfold reduction in the molecular weight of heavy asphaltene fractions (Kirkwood et al. 2005). This metabolic activity could result in an initial cleavage of the asphaltene structure into smaller fragments, starting the process of asphaltene biodegradation. Theoretical approaches to asphaltene biodegradation have been previously reported (Hernández-López et al. 2015); however, the specific metabolic pathways have not yet been described. Along with the metabolic capabilities mentioned above, the consortium was able to survive at least for 30 days in soil contaminated with 5000 mg asphaltenes kg^−1^, which was expected as each individual isolate was able to tolerate at least 60,000 mg asphaltenes kg^−1^. While the ability of bacteria to degrade hydrocarbons could not be directly associated with their tolerance to them, the use of highly tolerant isolates may increase the capacity of microbial consortia to improve the cometabolic breakdown of high-molecular-weight hydrocarbons (Montgomery et al. 2010), enhancing heavy PAH breakdown and microbial adaptation to soil (Kim et al. 2009).

The results presented here showed that highly tolerant bacteria, having the ability to use asphaltenes as the sole carbon source, have a better chance to adapt well to a highly polluted environment. When the developed consortium was inoculated in asphaltene-contaminated soils, it adapted and induced continuous CO_2_ production during biodegradation experiments, suggesting asphaltene mineralisation. The bacterial consortium developed in this study achieved higher biodegradation efficiencies (83 wt.%) and used high amounts of asphaltenes (10,000 mg asphaltenes kg^−1^ soil) compared to other studies evaluating the microbial degradation of asphaltenes in contaminated soils, in which maximum degradation efficiencies reached 79 wt.% in soils containing 140 mg kg^−1^ asphaltenes after 30 days (Yanto and Tachibana 2014b) or 86 wt.% degradation in soils containing 87 mg kg^−1^ asphaltenes after 30 days (Yanto and Hidayat 2020). High CO_2_ levels were detected during the first 5 days of treatment in soils inoculated with the consortium, then decreased to a minimum by day 35, and slowly increased until day 52. These results are consistent with previous studies reporting higher rates of mineralisation by microbial consortia at the initial stages of the degradation of high molecular weight PAHs in soil (EL-Saeid and Usman 2022; Zafra et al. 2014), which could be indicative of an initial degradation of the aliphatic moieties of asphaltenes along with soil organic matter, then reaching idiophase and secondary metabolite production that may favourably affect the degradation of higher weight asphaltene molecules. Further studies would be needed to corroborate this. The maximum CO_2_ production levels were obtained in non-contaminated control soils, indicating an absence of microbial growth inhibition caused by asphaltenes conducive to soil organic matter and sugarcane bagasse mineralisation. Several reports have demonstrated the positive effects of the addition of agroindustrial residues such as wheat bran (Barathi and Vasudevan 2003), wheat straw (Zhang et al. 2008), and sugarcane bagasse (Zafra et al. 2014) on the bioremediation of soils contaminated with different types of heavy hydrocarbons. The use of sugarcane bagasse has been demonstrated to improve the breakdown of heavy hydrocarbons serving as an alternative carbon source, thereby inducing cometabolism, in addition to acting as a soil texturizing agent and providing physical support for microbial growth (Fernández-Luqueño et al. 2011). In this work, both inoculated and non-inoculated soils amended with sugarcane bagasse were tested to reach maximum degradation rates of asphaltenes by a combination of the bacterial consortium and soil native microbiota or by only biostimulated native microbiota. As expected, asphaltene-contaminated soils inoculated with the bacterial consortium produced significantly higher amounts of CO_2_ at day 52 than the not-inoculated asphaltene-contaminated soils, resulting from asphaltene degradation exclusively by the microbial consortium. The observed differences in CO_2_ levels correlated with significant differences in asphaltene degradation efficiency, as bioaugmented and biostimulated soils reached up to 83 wt.% degradation out of 10,000 mg asphaltenes kg^−1^ after 52 days, in comparison to 63 wt.% achieved when only biostimulation was used. Despite the fact that both biostimulation plus bioaugmentation and biostimulation-only treatments have been demonstrated to be effective for the degradation of asphaltene in soils, the significant decrease in CO_2_ production in biostimulation-only treatments after 25 days of treatment compared to biostimulation plus bioaugmentation is indicative of the important role of sugarcane bagasse in supporting the active growth and long-term permanence of the introduced degrading consortium, significantly improving asphaltene degradation efficiency in highly polluted contaminated soils.

## Conclusion

In this study, a novel asphaltene-degrading bacterial consortium with remarkably high tolerance levels and high rates of asphaltene degradation was developed. This consortium could be used along with agroindustrial residues for the treatment of asphaltene pollution in soil and have adaptive advantages, enhancing its survival in asphaltene-contaminated soils due to its elevated tolerance and diverse catabolic potential, leading to efficient asphaltene mineralisation. This would be the first study to evaluate bacterial tolerance to extremely high asphaltene concentrations and microbial antagonism between individual degrading isolates, leading to the development of an improved degrading consortium for use in soil. This consortium is a promising candidate for the bioremediation of soil areas heavily contaminated with asphaltenes and other high-molecular-weight hydrocarbons, such as PAHs and petroleum resins, as well as for the treatment of aged soils containing complex mixtures of heavy hydrocarbons at different scales and by different approaches (e.g., landfarming or biopiles). The study of the specific cellular mechanisms that explain the high tolerance and degradation of asphaltenes is an important aspect of the consortium’s performance that requires further clarification. Hence, additional research using omics and other analytical tools would be needed to better explore the metabolic potential of the isolates composing the consortium and the specific mechanisms leading to asphaltene mineralisation and conferring their high tolerance levels, thus being able to relate structural changes in asphaltene molecules caused by microbial degradation to the abundance and expression of specific metabolic pathways and genes.

## Data Availability

No supplementary data or materials are available.

## References

[CR1] Al-Hawash AB, Dragh MA, Li S, Alhujaily A, Abbood HA, Zhang X, Ma F (2018). Principles of microbial degradation of petroleum hydrocarbons in the environment. Egypt J Aquat Res.

[CR2] Altschul SF, Gish W, Miller W, Myers EW, Lipman DJ (1990). Basic local alignment search tool. J Mol Biol.

[CR3] Arce-Ortega JM, Rojas-Avelizapa NG, Rodríguez-Vázquez R (2004). Identification of recalcitrant hydrocarbons present in a drilling waste-polluted soil. J Environ Sci Health A.

[CR4] ASTM (2022) Standard test method for determination of asphaltenes (heptane insolubles) in crude petroleum and petroleum products. Standard, vol ASTM D6560. ASTM. 10.1520/D6560-22

[CR5] Barathi S, Vasudevan N (2003). Bioremediation of crude oil contaminated soil by bioaugmentation of *Pseudomonas*
*fluorescens* NS1. J Environ Sci Health A.

[CR6] Bava YB, Geronés M, Giovanetti LJ, Andrini L, Erben MF (2019). Speciation of sulphur in asphaltenes and resins from Argentinian petroleum by using XANES spectroscopy. Fuel.

[CR7] Brown DM, Bonte M, Gill R, Dawick J, Boogaard PJ (2017). Heavy hydrocarbon fate and transport in the environment. Q J Eng Geol Hydrogeol.

[CR8] Chuah LF, Chew KW, Bokhari A, Mubashir M, Show PL (2022). Biodegradation of crude oil in seawater by using a consortium of symbiotic bacteria. Environ Res.

[CR9] Chuah LF, Nawaz A, Dailin DJ, Oloruntobi O, Habila MA, Tong WY, Misson M (2023). Investigating the crude oil biodegradation performance in bioreactor by using a consortium of symbiotic bacteria. Chemosphere.

[CR10] Chukwuma OB, Rafatullah M, Tajarudin HA, Ismail NA (2021). Review on bacterial contribution to lignocellulose breakdown into useful bio-products. Int J Environ Res Public Health.

[CR11] Cold Spring Harbor Laboratory (2010) M9 minimal medium (standard). 10.1101/pdb.rec12295

[CR12] EL-Saeid MH, Usman AR (2022). Influence of organic amendments and moisture regime on soil CO_2_-C efflux and polycyclic aromatic hydrocarbons (PAHs) degradation. Sustainability.

[CR13] Fernández-Luqueño F, Valenzuela-Encinas C, Marsch R, Martínez-Suárez C, Vázquez-Núñez E, Dendooven L (2011). Microbial communities to mitigate contamination of PAHs in soil - possibilities and challenges: a review. Environ Sci Pollut Res.

[CR14] Hernández-López EL, Ayala M, Vazquez-Duhalt R (2015). Microbial and enzymatic biotransformations of asphaltenes. Pet Sci Technol.

[CR15] Hii YS, Law AT, Chuah LF, Mohamed Shazili NA, Abdul Rashid MK, Yong JC (2009). Inhibitive chemical cue of *Pseudomonas*
*pseudoalcaligene* on biodegradation of anthracene in seawater medium. J Sustain Sci Manag.

[CR16] Jahromi H, Fazaelipoor MH, Ayatollahi S, Niazi A (2014). Asphaltenes biodegradation under shaking and static conditions. Fuel.

[CR17] Kim YM, Ahn CK, Woo SH, Jung GY, Park JM (2009). Synergic degradation of phenanthrene by consortia of newly isolated bacterial strains. J Biotechnol.

[CR18] Kirkwood KM, Ebert S, Foght JM, Fedorak PM, Gray MR (2005). Bacterial biodegradation of aliphatic sulfides under aerobic carbon- or sulfur-limited growth conditions. J Appl Microbiol.

[CR19] Koshlaf E, Ball A (2017). Soil bioremediation approaches for petroleum hydrocarbon polluted environments. AIMS Microbiol.

[CR20] Kshirsagar SD, Mattam AJ, Jose S, Ramachandrarao B, Velankar HR (2020). Heavy hydrocarbons as selective substrates for isolation of asphaltene degraders: a substrate-based bacterial isolation strategy for petroleum hydrocarbon biodegradation. Environ Technol Innov.

[CR21] Kuster E, Williams ST (1964). Selection of media for isolation of Streptomycetes. Nature.

[CR22] Lane DJ, Stackebrandt E, Goodfellow M (1991). 16S/23S rRNA sequencing. Nucleic acid techniques in bacterial systematics.

[CR23] Lavania M, Cheema S, Sarma PM, Mandal AK, Lal B (2012). Biodegradation of asphalt by *Garciaella*
*petrolearia* TERIG02 for viscosity reduction of heavy oil. Biodegradation.

[CR24] Lincoln SA, Hamilton TL, Valladares Juárez AG, Schedler M, Macalady JL, Müller R, Freeman KH (2015). Draft genome sequence of the piezotolerant and crude oil-degrading bacterium *Rhodococcus*
*qingshengii* strain TUHH-12. Genome Announc.

[CR25] Mansur AA, Adetutu EM, Kadali KK, Morrison PD, Nurulita Y, Ball AS (2014). Assessing the hydrocarbon degrading potential of indigenous bacteria isolated from crude oil tank bottom sludge and hydrocarbon-contaminated soil of Azzawiya oil refinery, Libya. Environ Sci Pollut Res.

[CR26] Mesbaiah FZ, Eddouaouda K, Badis A, Chebbi A, Hentati D, Sayadi S, Chamkha M (2016). Preliminary characterization of biosurfactant produced by a PAH-degrading *Paenibacillus* sp. under thermophilic conditions. Environ Sci Pollut Res.

[CR27] Montgomery MT, Boyd TJ, Osburn CL, Smith DC (2010). PAH mineralization and bacterial organotolerance in surface sediments of the Charleston Harbor estuary. Biodegradation.

[CR28] Nie H, Nie M, Diwu Z, Wang L, Yan H, Bai X (2021). Immobilization of *Rhodococcus*
*qingshengii* strain FF on the surface of polyethylene and its adsorption and biodegradation of mimic produced water. J Hazard Mater.

[CR29] Nzila A, Musa MM (2021) Current knowledge and future challenges on bacterial degradation of the highly complex petroleum products asphaltenes and resins. Front Environ Sci 9. 10.3389/fenvs.2021.779644

[CR30] Parthipan P, Elumalai P, Sathishkumar K, Sabarinathan D, Murugan K, Benelli G, Rajasekar A (2017). Biosurfactant and enzyme mediated crude oil degradation by *Pseudomonas*
*stutzeri* NA3 and *Acinetobacter*
*baumannii* MN3. 3 Biotech.

[CR31] Pineda-Flores G, Boll-Argüello G, Lira-Galeana C, Mesta-Howard AM (2004). A microbial consortium isolated from a crude oil sample that uses asphaltenes as a carbon and energy source. Biodegradation.

[CR32] Rohman MS, Pamulatsih E, Kusnadi Y, Yuwono T, Martani E (2015). An active of extracellular cellulose degrading enzyme from termite bacterial endosimbiont. Indones J Biotechnol.

[CR33] Shahebrahimi Y, Fazlali A, Motamedi H, Kord S, Mohammadi AH (2020). Effect of various isolated microbial consortiums on the biodegradation process of precipitated asphaltenes from crude oil. ACS Omega.

[CR34] Tamura K, Stecher G, Kumar S (2021). MEGA 11: molecular evolutionary genetics analysis version 11. Mol Biol Evol.

[CR35] Tavassoli T, Mousavi SM, Shojaosadati SA, Salehizadeh H (2012). Asphaltene biodegradation using microorganisms isolated from oil samples. Fuel.

[CR36] Ubani O, Atagana HI, Selvarajan R, Ogola HJ (2022) Unravelling the genetic and functional diversity of dominant bacterial communities involved in manure co-composting bioremediation of complex crude oil waste sludge. Heliyon 8:e08945. 10.1016/j.heliyon.2022.e08945 10.1016/j.heliyon.2022.e08945 PMC8857465 35243067

[CR37] Uche EC, Dadrasnia A, Heimann K, Karthikeyan OP, Muthu SS (2017). HC-0B-06: Biodegradation of hydrocarbons. Biodegradation and Bioconversion of Hydrocarbons.

[CR38] US-EPA (1996) Soil screening guidance: user’s guide. Superfund, vol EPA/540/R-96/018. United States Environmental Protection Agency, Washington D.C.

[CR39] Wang W, Yu Y, Dou Y, Wang JY, Sun C (2018). Species of family Promicromonosporaceae and family Cellulomonadeceae that produce cellulosome-like multiprotein complexes. Biotechnol Lett.

[CR40] Yanto DHY, Hidayat A (2020). Biodegradation of buried crude oil in soil microcosm by fungal co-culture. IOP Conf Ser: Mater Sci Eng.

[CR41] Yanto DHY, Tachibana S (2014). Enhanced biodegradation of asphalt in the presence of Tween surfactants, Mn2+ and H2O2 by *Pestalotiopsis* sp. in liquid medium and soil. Chemosphere.

[CR42] Yanto DHY, Tachibana S (2014). Potential of fungal co-culturing for accelerated biodegradation of petroleum hydrocarbons in soil. J Hazard Mater.

[CR43] Yetti E, Thontowi A, Yopi Y (2016). Polycyclic aromatic hydrocarbon degrading bacteria from the Indonesian marine environment. Biodiversitas.

[CR44] Zafra G, Absalón ÁE, Cuevas MDC, Cortés-Espinosa DV (2014). Isolation and selection of a highly tolerant microbial consortium with potential for PAH biodegradation from heavy crude oil-contaminated soils. Water Air Soil Pollut.

[CR45] Zafra G, Taylor TD, Absalón AE, Cortés-Espinosa DV (2016). Comparative metagenomic analysis of PAH degradation in soil by a mixed microbial consortium. J Hazard Mater.

[CR46] Zafra G, Absalón Á, Anducho-Reyes M, Fernandez FJ, Cortés-Espinosa DV (2017). Construction of PAH-degrading mixed microbial consortia by induced selection in soil. Chemosphere.

[CR47] Zhang K, Hua XF, Han HL, Wang J, Miao CC, Xu YY, Huang ZD, Zhang H, Yang JM, Jin WB, Liu YM, Liu Z (2008). Enhanced bioaugmentation of petroleum and salt-contaminated soil using wheat straw. Chemosphere.

[CR48] Zhang D-C, Liu H-C, Zhou Y-G, Schinner F, Margesin R (2011). *Tistrella*
*bauzanensis* sp. nov., isolated from soil. Int J Syst Evol Microbiol.

